# Bystander interventions against gender-based violence and harassment in the workplace: a scoping review

**DOI:** 10.3389/fpsyg.2025.1570812

**Published:** 2025-05-29

**Authors:** Kristan Stampe Nielsen, Maj Hansen, Eva Gemzøe Mikkelsen

**Affiliations:** THRIVE, Department of Psychology, University of Southern Denmark, Odense, Denmark

**Keywords:** sexual harassment, gender-based violence and harassment, bystander intervention, workplace harassment, primary prevention

## Abstract

Gender-based violence and harassment (GBVH) has detrimental effects on organisational performance and employee well-being. Primary-level bystander intervention programmes that enable employees to act when witnessing GBVH offer a promising prevention strategy. Despite the increased focus on the importance of primary-level bystander interventions, limited efforts have been made to systematically synthesise research in this area. The present scoping review therefore sought to consolidate research on primary-level bystander interventions implemented in organisations by exploring the theoretical frameworks used to develop these interventions, determining outcomes, and examining facilitators and obstacles for their implementation. Five databases [Embase (Ovid), MEDLINE (Ovid), Scopus, Web of Science, and PsycINFO (Ovid)], and grey literature were systematically searched. Out of 11.302 references screened, 14 studies were included in the present scoping review. Findings suggest that primary-level bystander interventions, particularly bystander training workshops incorporating practice exercises, can increase prosocial bystander behaviour. However, the paucity of follow-up measurements makes it challenging to evaluate their long-term efficacy. The included studies underscore the importance of tailoring interventions to specific workplace contexts. While comprehensive program theories underpin most studies, organisational change theories were typically absent, even when interventions aimed to achieve cultural change. In conclusion, this review emphasises the significance of bystander interventions for primary GBVH prevention and underscores the need for further research to identify optimal practices and ensure enduring effectiveness.

**Systematic review registration:**

The protocol is registered on the Open Science Framework website at https://osf.io/3pt5k/.

## 1 Introduction

Gender-based violence and harassment (GBVH) in the workplace is a global issue impacting millions’ health and well-being (Li et al., 2023) and costing organisations billions (McDonald, 2012; Deloitte Access Economics, 2019). GBVH at work is defined as “violence and harassment directed at persons because of their sex or gender, or affecting persons of a particular sex or gender disproportionately, and includes sexual harassment” (International Labour Organisation, 2019, article 1, b).

Negative individual consequences of GBVH include depression (Friborg et al., 2017), self-harm (Jin et al., 2018), suicide or suicide attempts (Magnusson Hanson et al., 2020), and posttraumatic stress disorder (Willness et al., 2007). At an organisational level, GBVH may result in long-term sickness absence (Blindow et al., 2021), higher turnover rates, reduced effectiveness, low morale, and poor corporate reputation (Willness et al., 2007; Henning et al., 2017). Although GBVH can happen to anyone, women are victimised at a much higher rate than males (UN Women and International Labour Organization, 2019) and young women, women from ethnic or sexual minorities, and migrant workers have an increased risk of exposure to GBVH at their workplace (Berdahl and Moore, 2006; Shaw et al., 2018). Other risk factors include being a woman in a traditionally male-dominated profession (McLaughlin et al., 2017), unequal power relations (National Academies of Sciences, Engineering, and Medicine, 2018), and precarious work (Shaw et al., 2018). The disproportionate prevalence for women and minorities may be explained using the intersectionality framework (Crenshaw, 1991), which highlights how multiple marginalised identities (e.g., race, disability, sexuality) interact in complex ways, amplifying risk, discrimination, and severity of consequences (Bondestam, 2024).

Given its potentially grave consequences, prevention of GBVH is of utmost importance. Yet, little is known about which strategies, policies, interventions, and training programmes are effective in preventing GBVH in the workplace (Campbell and Chinnery, 2018; Diez-Canseco et al., 2022). During the last decade, mobilising bystanders have received increased attention as an avenue for preventing workplace mistreatment (Hershcovis and Barling, 2010; Mulder et al., 2014; Hershcovis et al., 2017; Mikkelsen and Høgh, 2019; Vranjes et al., 2021). Recently, a growing body of research has demonstrated that bystanders may also play an important role in GBVH prevention. Bystanders can prevent the development of GBVH by challenging discriminatory behaviours, e.g., by interrupting sexist jokes (Banyard et al., 2004, 2021; Cares et al., 2015), by directly intervening in situations with GBVH (Labhardt et al., 2017), and by giving support to targets following their exposure to GBVH (Rowe, 2018). Together, such behaviour is classified as prosocial behaviours, which stands in contrast to antisocial behaviours such as joining in on harassment or laughing along. This said, research also shows that many bystanders choose not to intervene at all and remain passive in situations involving GBVH (McDonald et al., 2016). Given the potentially important role of bystanders in preventing GBVH, practitioners and researchers are increasingly exploring both bystander interventions and specific bystander behaviours. The term ‘bystander intervention’ can be confusing, as it can be used to describe both the actions taken by individual bystanders and programmes or training developed to educate people on bystander behaviour used to intervene in GBVH situations. In this article, “bystander intervention” refers to workplace programmes aimed at preventing GBVH, while “bystander behaviours” or “bystander actions” denote the responses of individual bystanders.

Bystander interventions span three levels. Primary-level interventions work preventively by addressing cultural and structural antecedents, altering attitudes, values, and beliefs to foster protective conditions (e.g., increased bystander behaviours) and reduce risk factors (e.g., acceptance of sexualised humour). Secondary interventions engage directly with high-risk situations, and tertiary interventions support targets and sanction offenders to deter future incidents (Larcombe, 2014; Mainwaring et al., 2022). Existing research and reviews of bystander interventions have either focused on other types of mistreatment (e.g., sexual violence (Jouriles et al., 2018), bullying (Pouwelse et al., 2021)) or on GBVH interventions in higher education (Labhardt et al., 2017; Bondestam and Lundqvist, 2020; Mujal et al., 2021) and on individual outcomes, such as increased knowledge and improved attitudes towards GBVH prevention in this context (DeGue et al., 2014; Fenton et al., 2016; Labhardt et al., 2017), with little attention being paid to contextual and organisational factors. As such, there is a limited understanding of how primary-level bystander interventions function in other settings, such as workplaces, and which theoretical frameworks should support them. From a research and practice perspective, it is essential to map out the development, implementation, and evaluation of bystander interventions in various workplace contexts. When evaluating organisational interventions, it is therefore crucial to assess their theoretical background, as this influences both content and implementation methods (Lassiter et al., 2021). Indeed, Bartholomew and Mullen (2011) argue that suitable theories are paramount when developing interventions and that researchers should deliver detailed reports of such. This includes describing the theories on which the intervention is based, the proposed change mechanism, and the mediating and moderating factors expected to influence implementation. In addition, K. Nielsen and Abildgaard (2013) argue that contextual factors are as important to study as effects, given their influence on implementation.

This scoping review is the first to explore primary-level bystander interventions implemented in workplaces. Specifically, it aims to (1) map and evaluate the design, implementation, and outcomes of these interventions, (2) explore the theoretical frameworks that inform their development, and (3) analyse context factors that may facilitate or obstruct their implementation. By addressing gaps in existing literature, this review provides actionable insights that might inform both intervention development and implementation practices.

## 2 Methods

Unlike systematic reviews, which synthesise evidence to answer specific questions about intervention efficacy, scoping reviews address broad inquiries and compare diverse evidence. This makes them ideal for examining emerging topics and identifying insights from existing literature, especially in underexplored areas (Peters et al., 2020; Munn et al., 2022). Although not mandatory, scoping reviews may assess and discuss the quality of included studies when necessary (Munn et al., 2018; Peters et al., 2022). In alignment with the aim of the current study, we have included such an assessment and discussion. A protocol was developed based on the Joanna Briggs Institute’s ‘best practice’ guidelines (Peters et al., 2020, 2022) and registered at Open Science Framework (Nielsen et al., 2023) before the search was initiated.

### 2.1 Search strategy

The following databases were systematically searched on January 10th, 2023, and again on August 15th, 2024: Embase (Ovid), MEDLINE (Ovid), Scopus, Web of Science, and PsycINFO (Ovid). Additionally, grey literature was searched using search engines (e.g., Google, Google Scholar, and Semantic Scholar), consulting subject matter experts, government agency websites (e.g., OurWatch, Australia), university research group websites (e.g., Prevention Innovations Research Center, University of New Hampshire), and reference chaining from included studies. The search strategy for this scoping review followed the 15 steps outlined by Bramer et al. (2018). Search terms were identified through preliminary searches in PsycINFO and Embase on “sexual harassment,” “interventions,” “prevention,” “gender-based violence and harassment” and “workplace” or “organisation” noting key concepts and terms from eligible papers and reviews on similar topics. The final search string included 15 related terms (e.g., sexual harassment, gender discrimination, sex-based bullying) and was evaluated by a research librarian for balance between specificity and sensitivity (Bramer et al., 2018). As an additional quality control measure results were scanned for predefined key studies. Complete search strings and parameters are available in Supplementary material S1.

### 2.2 Eligibility criteria

The eligibility criteria were based on the ‘Population, Concept, Context’ framework (Peters et al., 2020). Studies were eligible if they addressed the primary prevention of GBVH by employing bystander interventions in workplace settings, including cases where these interventions were part of larger projects. Multi-purpose interventions (i.e., interventions focusing on reducing both domestic and work-related GBVH) was considered on a case-by-case basis. Workplaces are understood as a place of employment, and as such places of education can be included if the intervention targets employees and not students. Prevention efforts are broadly understood and include employee workshops, seminars, lectures, online training etc. To be included, studies had to report outcome measures, expected outcomes, or a testable intervention framework, focusing on internal GBVH (i.e., between co-workers or in manager-subordinate relationships). Studies were excluded if interventions were only at a policy level, focused on specific incidents, increasing report rates, handling incidents of GBVH afterwards, or supporting victims. All types of empirical studies published in peer-reviewed journals, intervention study protocols, and grey literature (e.g., reports, dissertations, government agency interventions) in English or Scandinavian languages (Danish, Swedish, Norwegian) were included. A more detailed description is available in the protocol (Nielsen et al., 2023).

### 2.3 Study selection

**Figure 1:**
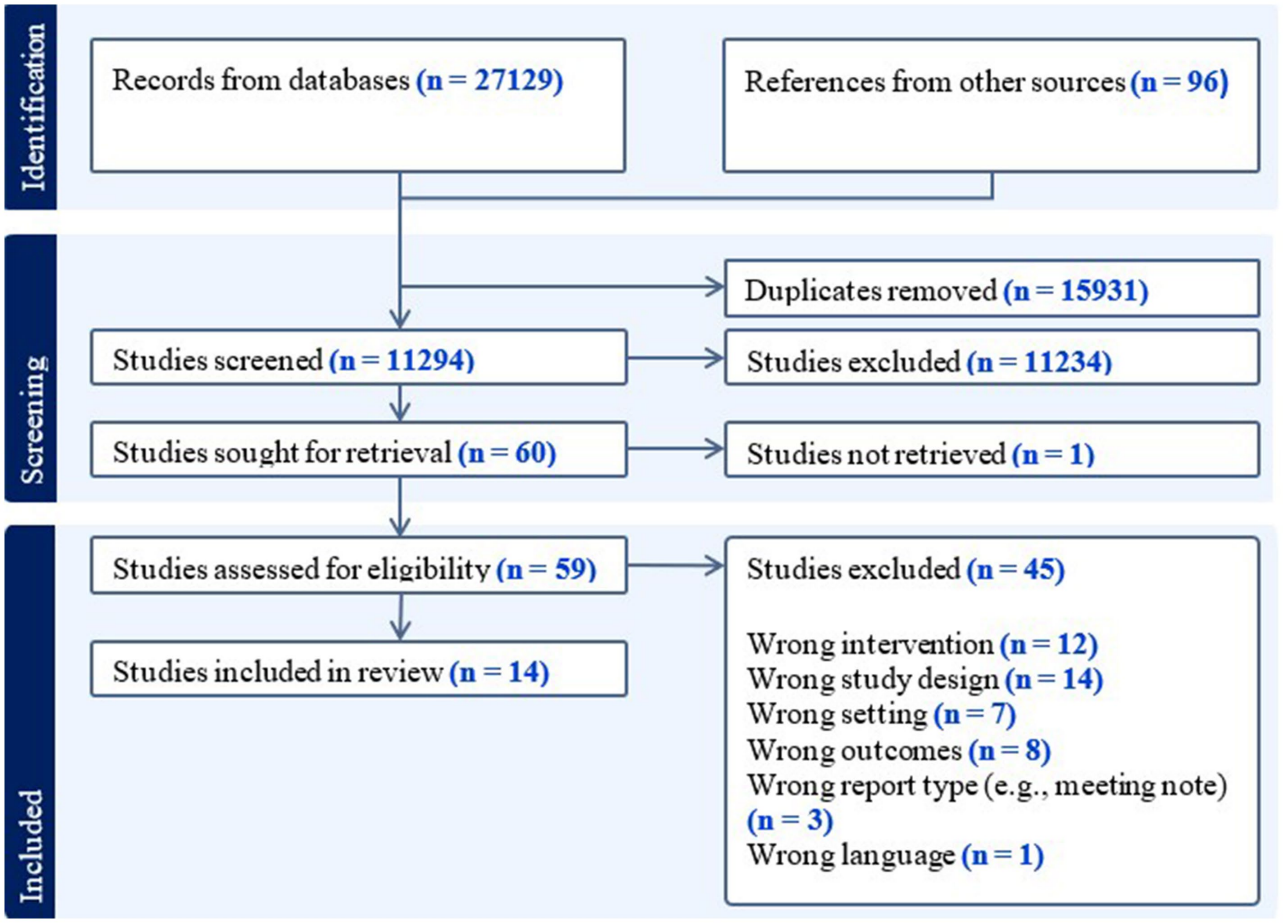
Flowchart of the screening process.

During the first round in January 2023 13,966 records were retrieved, reduced to 9,164 after removing duplicates using Covidence systematic review software (Veritas Health Innovation, 2023). Fifty-five studies were included for full-text review, but only 54 studies were read, as one could not be retrieved (Biles, 1981). Of these, 13 studies were included in the present review. During the second round of updating the searches in August 2024, another 2,138 records were added after duplicate removal. Five of these studies were full-text screened, with one study being included. Title-abstract and full-text screening were conducted by the first author with the help of a student assistant in round one and a research assistant in round two. Conflicts were resolved through discussion. Two records were reviewed and discussed with the third author. One study (Bell et al., 2002) was removed after screening; initially included under ‘testable intervention framework,’ it was reclassified as a theoretical framework and excluded after discussion among the authors. See Figure. 1 for the selection process.

### 2.4 Data extraction

A data extraction form (see Supplementary material S1) was designed for this scoping review to extract data on study characteristics, aims, intervention characteristics, participants, and outcome measures. A full table of the included studies and their characteristics is available in Supplementary material S2. This review reports both a narrative synthesis and a quantitative summary of the included studies. We did not assess the risk of bias, as this is not standard practice for scoping reviews (Tricco et al., 2016; Peters et al., 2020).

## 3 Results

This section provides an overview of the included studies, summarised in Table 1. Interventions were categorised as successful, partially successful, or non-successful. An intervention was deemed successful if it achieved a significant increase in all or most bystander-related outcomes, partially successful if there was an increase in some of the outcomes, and non-successful if it showed no increases in bystander-related outcomes. This categorisation highlighted the variability in outcomes and identified common factors associated with different levels of success. Using this categorisation, we aimed to provide a nuanced understanding of the factors influencing implementation and outcomes of the interventions. We did not find any noticeable differences between studies reported in grey literature and peer-reviewed sources, though the number of references from outside databases was very limited.

**Table 1:** Study and intervention characteristics–summarised.

Characteristic	Number of Studies
Intervention aim*	
Improving awareness, knowledge, and attitudes	14
Empower bystanders to intervene	13
Organisational and/or cultural change/transformation	9
Policy development and implementation	7
Theoretical foundation*	
Violence prevention and bystander intervention theories	9
Gender, diversity, and intersectionality theories	7
Social, learning and capacity building theories	6
Public health and economic empowerment theories	3
Organisational and team theories	3
No theoretical foundation or none reported	2
Intervention and change theory	1
Scales and measurements*	
Bystander related measures (e.g., bystander behaviour, bystander intention to act etc.)	6
Experiencing and/or witnessing sexual harassment and/or violence measures	4
Custom developed scales (i.e., not independently tested)	4
Measures of the acceptance of sexual harassment and/or violence	4
Qualitative assessments (e.g., interviews and observation)	4
General knowledge, perceptions, and attitudes about sexual harassment scales	4
Gender attitudes and equality measures	3
Workplace climate and workplace culture measures	3
Specific intervention training effectiveness measures	3
Measures of psychological impact and coping strategies	2
Incidence report measure	1
Intervention type*	
Bystander training workshop	9
Larger organisational change effort	5
Lecture/seminar	5
Changes to company policies	5
Information campaign	4
Art performance	1
Intervention design	
Complex multi-component interventions	6
Single-session interventions	6
Multi-session interventions (4–16)	2
Content delivery*	
In person presentation/workshop	10
Passive communication (i.e., posters)	3
Video presentation	3
Theatre performance	1
Online presentation/workshop	1
Not reported	2
Target audience*	
Employees	8
Whole organisation	3
Middle management	3
Soldiers	2
Other^a^	1
Not reported	1
Was the intervention effective?	
Yes	7
Partially^b^	5
No	1
Not applicable^c^	1
Was the effect retained over time?	
Yes^d^	1
Partially^e, f, g, h, i^	4
No follow-up was done	6
Not applicable^j^	2
Individual outcomes*^, c^	
Positive changes in attitudes and/or knowledge about sexual harassment	11
Increase of positive bystander behaviours or intentions	7
Negative results (e.g., backlash effects)	2
No results	1
Reduction in perpetrator propensity	1
Organisational outcomes*^, c^	
Positive changes in organisational culture and climate (e.g., greater gender equity)	5
Changes in organisational policies and procedures	4
Changes in sexual harassment prevalence	2
No results	7
Negative results	1
Facilitating factors*	
Leadership commitment and support	4
Framing men as allies instead of perpetrators	3
Using adaptable programs that could be tailored to the organisation	4
Using an inclusive and participative approach	3
None reported	5
Obstructing factors*	
Delivery and facilitation challenges	4
Insufficient resources and support	3
Organisational factors	3
Cultural and political factors	2
Lack of experience and training	2
Resistance, backlash, and interference	2
None reported	6

* = categories are not exclusive, thus the total number of studies in each category can exceed 14; a = Students, campus communities and sports clubs; b = Inferential statistics not reported in all studies; c = Naved et al. (2021) have been excluded because the results are unreliable owing to data contamination; d = One-month follow-up; e = 4,5-month follow-up; f = One-year follow-up; g = Evaluation of program effect 15 years later; h = 6–12 month follow-up interview; i = 1–2 month follow-up; j = Naved et al. (2021) and Sarpy et al. (2022).

### 3.1 Study characteristics

The present review includes 14 empirical studies: 11 studies from Western, Educated, Industrialised, Rich, Democratic (WEIRD) cultures (Australia, USA, and Europe) (Jacobs et al., 2000; Bingham and Scherer, 2001; Durey, 2011; Potter and Moynihan, 2011; Foubert and Masin, 2012; Crisp and Taket, 2018, 2023; Fischer et al., 2021; Martini and De Piccoli, 2021; Sarpy et al., 2022; Cronin et al., 2024), two studies from Bangladesh (Stuart et al., 2017; Naved et al., 2021), and one study with participants from Africa, Asia, Caribbean, and the Pacific region (Kuppuswami and Ferreira, 2022). Most studies (*n* = 12) have been published after 2011, seven of these after 2021 (Fischer et al., 2021; Martini and De Piccoli, 2021; Naved et al., 2021; Kuppuswami and Ferreira, 2022; Sarpy et al., 2022; Crisp and Taket, 2023; Cronin et al., 2024). The studies were conducted in diverse organisational contexts: three studies (Jacobs et al., 2000; Bingham and Scherer, 2001; Martini and De Piccoli, 2021) were in a higher education setting, two studies (Potter and Moynihan, 2011; Foubert and Masin, 2012) used military personnel, two (Stuart et al., 2017; Kuppuswami and Ferreira, 2022) were in non-profit organisations, two (Durey, 2011; Naved et al., 2021) used blue-collar workers, two (Crisp and Taket, 2018; Sarpy et al., 2022) used white-collar workers in municipalities, two was done in research agencies (Fischer et al., 2021; Cronin et al., 2024), and the last used a mix of different workplaces (Crisp and Taket, 2023).

The number of participants varied from eight to 800, with some studies having high dropout rates (13–80%), though dropout rates could not be calculated for all studies. Twelve studies included both male and female participants, with one targeting only men (Foubert and Masin, 2012) and one targeting only women (Naved et al., 2021).

Nine studies used a non-randomised experimental study design (Bingham and Scherer, 2001; Potter and Moynihan, 2011; Crisp and Taket, 2018, 2023; Fischer et al., 2021; Martini and De Piccoli, 2021; Naved et al., 2021; Kuppuswami and Ferreira, 2022; Cronin et al., 2024), the remaining five used various study designs: action research (Durey, 2011), mixed-method study (Stuart et al., 2017), interview study (Sarpy et al., 2022), one a randomised control trial (Foubert and Masin, 2012), and the last used a repeated cross-sectional design (Jacobs et al., 2000). Over 30 different instruments measuring both outcomes and associated variables were identified, wherefore they were aggregated into larger concept categories, as shown in Table 1. Due to the diversity of outcome measures, results were also aggregated for both individual and organisational outcomes. See Supplementary material S2 for Table 1 with citations and a sortable characteristics table.

Seven studies (Jacobs et al., 2000; Durey, 2011; Crisp and Taket, 2018, 2023; Fischer et al., 2021; Martini and De Piccoli, 2021; Kuppuswami and Ferreira, 2022) did not use a comparator in outcome evaluation, four studies (Bingham and Scherer, 2001; Potter and Moynihan, 2011; Naved et al., 2021; Cronin et al., 2024) used passive comparators, and one (Foubert and Masin, 2012) used an active comparator. For two studies (Stuart et al., 2017; Sarpy et al., 2022) this criterion was not applicable.

### 3.2 Intervention descriptions

Interventions were divided into the categories of successful interventions (SI), partially successful interventions (PSI), or non-successful interventions (NSI). The 14 interventions are briefly described below. As the aims of the reviewed interventions overlap significantly, they were grouped into four categories (see Table 1). See Supplementary material S2 for further details on each study and their intervention.

#### 3.2.1 Successful interventions (SI)

Six of seven successful studies used bystander intervention training workshops as the main or sole content delivery method (Jacobs et al., 2000; Durey, 2011; Potter and Moynihan, 2011; Foubert and Masin, 2012; Stuart et al., 2017; Cronin et al., 2024). Three offered the workshops as single-session, stand-alone interventions (Potter and Moynihan, 2011; Foubert and Masin, 2012; Cronin et al., 2024), while three studies incorporated the workshops within broader organisational intervention strategies (Jacobs et al., 2000; Durey, 2011; Stuart et al., 2017).

Despite differences, all seven studies showed a high degree of intervention customisation to their respective settings. Three studies adapted American college campus interventions to fit the military (Potter and Moynihan, 2011; Foubert and Masin, 2012) or Australian workplaces (Crisp and Taket, 2023). Cronin et al. (2024) developed a new ‘fit for purpose’ intervention. The remaining three (Jacobs et al., 2000; Durey, 2011; Stuart et al., 2017) tailored their interventions to align with their specific organisational change projects.

##### 3.2.1.1 Single-session stand-alone bystander interventions

Foubert and Masin (2012) tested an adapted version of ‘The Men’s Program’, a one-hour, men-only workshop, focusing on developing empathy with victims of GBVH, survivor support strategies, and modelling proactive, socially responsible bystander behaviour. Potter and Moynihan (2011) adapted ‘Bringing in The Bystander’ to a military context. The mixed-gender 4.5-h workshop aimed to educate soldiers about sexual violence and stalking, promote active bystander behaviours, empower participants with intervention skills, and stress communal responsibility in decreasing sexual violence and harassment. Cronin et al. (2024) tested the ‘Building a Better Fieldwork Future’ intervention, a peer-facilitated 90-min interactive training session addressing sexual harassment and assault in field science settings. The training session included five major components, four focus areas (components 1–4) and the use of scenario-based group discussion (component 5): (1) Definitions of sexual harassment and assault and why fieldwork is a high-risk setting. (2) How to prepare for fieldwork with best practises for protocols and codes of conduct. (3) Information on basic bystander action. (4) Introduction to the internal reporting process. (5) The scenario-based discussion was used throughout the session, with cases and discussions related to the current topic.

##### 3.2.1.2 Complex interventions using bystander training

Durey (2011, 2023 personal communication) used a single-session training scheme, Stuart et al. (2017) employed a multi-session program, while Jacobs et al. (2000) did not report any details about duration, type or content of the workshops. In Durey (2011), bystander workshops were part of a larger organisational change project aimed at preventing violence against women at home and in the workplace. The intervention included a 30-min mixed-gender training on domestic and workplace violence, reasons for staying in abusive relationships, bystander tools, and practice exercises. The project also introduced a new policy on sexual harassment and violence, an internal information campaign, and a media strategy with promotional materials and press releases. Stuart et al. (2017) evaluated the organisational transformation project ‘Gender Quality Action Learning programme’ (GQAL). This was implemented by the non-profit organisation BRAC between 1994 and 2003 and reached over 16,000 employees in total. The programme featured action learning workshops on gender inequality, gendered violence, personal and organisational change, bystander behaviour, male allyship, and action plan development for workplace gender issues. The exact number of sessions varied as different worksites chose different issues.

##### 3.2.1.3 Bystander intervention as theatre performance

Crisp and Taket (2023) tested a 30-min theatre performance in different settings. In this, a single male actor portrayed four different male bystanders with different bystander behaviours. This was followed by a moderated post-performance panel discussion featuring local agencies engaged in sexual harassment and violence prevention.

#### 3.2.2 Partially successful interventions (PSI)

Only two of the five PSI used bystander training workshops as their main delivery method (Crisp and Taket, 2018; Fischer et al., 2021). Fischer et al. (2021) employed a single 2-h workshop facilitated by a peer educator (research team leader), which aimed to teach participants how to identify, address, and prevent sexual harassment during a field research campaign. Conversely, Crisp and Taket (2018) outlined a complex organisational change project that included a multi-channel information campaign, policy changes, and workshops on bystander behaviours. However, as the original project files are under permanent embargo, it is unclear whether they used single-or multi-session workshops or what the content of these was.

All five PSI were highly customised. Kuppuswami and Ferreira (2022) and Crisp and Taket (2018) conducted needs assessments before developing their interventions. Fischer et al. (2021) adapted the bystander training workshop multiple times based on participant feedback. The last two interventions (Bingham and Scherer, 2001; Martini and De Piccoli, 2021) were also tailored to their respective organisational contexts.

##### 3.2.2.1 Single and multi-lecture interventions

The other three partially successful studies used either single-session (Bingham and Scherer, 2001) or multi-session lectures (Martini and De Piccoli, 2021; Kuppuswami and Ferreira, 2022) as their primary delivery methods. Bingham and Scherer (2001) tested a 30-min lecture that included a videotaped speech by the school chancellor, a briefing on sexual harassment definitions and workplace policies, and a discussion session. In Kuppuswami and Ferreira’s (2022) study, participants attended six online lectures (no duration reported) and completed 10 unspecified “hands-on application of concepts and strategies” (Kuppuswami and Ferreira, 2022, p. 398) assignments and nine organisational workshops for assignment review. The modules covered Gender Equality Concepts, Women’s Human Rights, The Concept of Power, Gender Analysis and Strategic Planning, Gender Concerns in Humanitarian Crises, and Gender-Responsive Programming. Martini and De Piccoli (2021) used two plenary sessions and two small group lectures. These explored gender issues, sexual violence and harassment, discrimination against women, and legal frameworks, using dynamic learning tools such as case studies, films, and theoretical discussions on sexism, stereotypes, and bystander behaviour. However, these were not considered ‘bystander training workshops’ as they lacked practical application or training of behaviours, offering only theoretical discussions.

#### 3.2.3 Non-successful interventions (NSI)

Both NSI (Naved et al., 2021; Sarpy et al., 2022) used complex multicomponent interventions, which included bystander training workshops. Naved et al. (2021) attempted to implement an intervention consisting of; bystander training workshops for female workers; dialogue meetings between female workers, managers, and male workers about sexual harassment and violence in the workplace; an anti-violence communication campaign; and discussions on female-friendly company policies.

Sarpy et al. (2022) attempted to implement a multicomponent intervention project consisting of “at least seven evidence-based interventions, such as bystander training, policy changes, and providing resources and education” (Sarpy et al., 2022, p. 2). As they did not elaborate on the individual components, the bystander training component cannot be evaluated. The factors identified as contributing to the lack of success in these studies will be discussed in the section on obstructing factors.

### 3.3 Theories and frameworks behind the interventions

When evaluating the theoretical foundation of interventions, it is important to differentiate between the theories used for their development and the theories taught during the intervention. This section focuses on the former. Across 12 studies (Durey, 2011; Potter and Moynihan, 2011; Foubert and Masin, 2012; Stuart et al., 2017; Crisp and Taket, 2018, 2023; Fischer et al., 2021; Martini and De Piccoli, 2021; Naved et al., 2021; Kuppuswami and Ferreira, 2022; Sarpy et al., 2022; Cronin et al., 2024), we identified over 25 different theories, frameworks, and methodologies, whereas two studies (Jacobs et al., 2000; Bingham and Scherer, 2001) did not report any guiding theory. We aggregated these into broader categories, as shown in Table 1. For brevity, this section is structured according to the SI, PSI and FI categories and reports only the primary theories which inspired the development of the interventions (see Supplementary material S2 for further details).

#### 3.3.1 SI

One study (Jacobs et al., 2000) did not report the theories which might have been used to develop their intervention programme or the bystander workshops. Three studies (Potter and Moynihan, 2011; Foubert and Masin, 2012; Crisp and Taket, 2023) primarily drew on existing bystander intervention programs (You The Man, The Men’s program, Bringing in the Bystander) and their underlying theories. Two studies (Durey, 2011; Cronin et al., 2024) drew more generally on research on bystander intervention and bystander behaviours (e.g., Banyard, 2008; Banyard et al., 2009): Durey (2011) also included theories on gendered violence (e.g., Berkowitz, 2002), and organisational theory, while Cronin et al. (2024) included adult learning theory and intervention and change theories. Three other studies (Durey, 2011; Potter and Moynihan, 2011; Crisp and Taket, 2023) also utilised social norms and adult learning theories. One study (Stuart et al., 2017) mainly used gender transformation theory, organisational diagnostics, organisational development theory, and Freirean pedagogy principles.

#### 3.3.2 PSI

When analysing PSI, we find that more diverse theories are employed, and one study (Bingham and Scherer, 2001) that does not report any theoretical foundation. Similar to most SI, two PSI (Crisp and Taket, 2018; Fischer et al., 2021) were informed by theories on bystander behaviour. Fischer et al. (2021) also used Quick and McFadyen’s (2017) work and high-performance team theory, supplemented with unspecified bystander training material.

Theories on gender and gender differences were used to develop the interventions in three studies (Crisp and Taket, 2018; Martini and De Piccoli, 2021; Kuppuswami and Ferreira, 2022). One study (Martini and De Piccoli, 2021) was based solely on gender system justification theory. Kuppuswami and Ferreira (2022) used a constructivist theory of learning and gender assessment frameworks from international agencies to develop their intervention.

Crisp and Taket (2018) drew on a broad range of theories, including gender theory, asset-based violence prevention, intersectionality, and social norms theory. No information was provided on which kind of bystander theories were utilised.

#### 3.3.3 NSI

Of the NSI, Sarpy et al. (2022) used the comprehensive ‘Change the Story: A Shared Framework for Primary Prevention of Violence against Women in Australia’ (Our Watch, 2021) and the socio-ecological model of interventions. This framework incorporates theories from multiple disciplines, including theories on bystander behaviour, intersectionality, gendered violence theory, and violence and harassment prevention theory.

Naved et al.’s (2021) intervention was based on a comprehensive programme theory from an interview study (Naved et al., 2018), detailed in the study protocol (Mamun et al., 2018). The key theoretical pillars were women’s economic empowerment, gender transformation theory, and theories on the cultural, social, and structural antecedents of GBVH in the workplace.

### 3.4 Outcomes and retention of effect

In the following sections, we first present the individual outcomes, then the organisational outcomes and finally, we examine whether the outcomes were retained. The study by Naved et al. (2021) is excluded due to unreliable data, and the Sarpy et al. (2022) study is excluded from the individual outcomes section due to a lack of individual measurements.

#### 3.4.1 Individual outcomes

##### 3.4.1.1 Increase of knowledge

Of the eligible 12 studies, seven reported a significant increase in participants’ knowledge about sexual harassment (Bingham and Scherer, 2001; Durey, 2011; Stuart et al., 2017; Fischer et al., 2021; Martini and De Piccoli, 2021; Kuppuswami and Ferreira, 2022; Cronin et al., 2024), though the types of knowledge tested varied. Bingham and Scherer (2001) measured knowledge of legal and policy aspects. Three studies (Durey, 2011; Fischer et al., 2021; Martini and De Piccoli, 2021) focused on areas such as prevalence, forms of violence and harassment, and risk groups. Two studies (Stuart et al., 2017; Kuppuswami and Ferreira, 2022) assessed knowledge of gender equality, women’s human rights, and gender power dynamics. The last study focused on knowledge of “existing resources to … prevent, intervene in, and report sexual harassment” (Cronin et al., 2024, p. 3).

##### 3.4.1.2 Changes in individual attitudes towards GBVH

Five studies (Jacobs et al., 2000; Durey, 2011; Foubert and Masin, 2012; Stuart et al., 2017; Kuppuswami and Ferreira, 2022) indicated significant positive changes in attitudes towards gender, sexual harassment, and gender equity. Three studies (Jacobs et al., 2000; Durey, 2011; Kuppuswami and Ferreira, 2022) measured this as an increase in gender positivity or a decrease in gender insensitivity. Two studies (Foubert and Masin, 2012; Martini and De Piccoli, 2021) found a significant reduction in rape myth acceptance, though Martini and De Piccoli (2021) only found significant reductions for two out of four rape myths. A positive, albeit non-significant, change in gender attitudes was also noted by Crisp and Taket (2018). Three studies found (Foubert and Masin, 2012; Stuart et al., 2017; Crisp and Taket, 2023) significant improvements in participants’ perceptions of the severity of sexual harassment.

##### 3.4.1.3 Increase in bystander behaviour and intentions

Multiple studies showed an increase in bystander-related measurements, such as intention, efficacy, and behaviours. However, only two studies (Potter and Moynihan, 2011; Crisp and Taket, 2023) measured the number of prosocial bystander behaviours performed. Potter and Moynihan (2011) found that participants performed more bystander behaviours towards acquaintances and strangers than non-participants. Crisp and Taket (2023) noted an increase in bystander intentions, but not bystander behaviour. Five studies (Durey, 2011; Potter and Moynihan, 2011; Foubert and Masin, 2012; Fischer et al., 2021; Cronin et al., 2024) demonstrated a significant improvement in bystander intention to intervene, however in the Cronin et al. (2024) study this was only for within-subject measures. There were no significant differences between the intervention and control groups. Two studies (Foubert and Masin, 2012; Cronin et al., 2024) found a significant increase in bystander self-efficacy, while Martini and De Piccoli (2021) found no significant post-intervention changes. Finally, qualitative data from Stuart et al. (2017, p. 70) provide anecdotal evidence of increased bystander behaviours.

##### 3.4.1.4 Backlash effects

Two studies (Bingham and Scherer, 2001; Fischer et al., 2021) reported negative outcomes alongside positive findings, related to male participants’ perceptions of the workshop material. Bingham and Scherer (2001, p. 125) found that male participants were less likely to see coercive sexual harassment as a problem, less willing to report it, and more prone to victim-blaming. Fischer et al. (2021, p. E2145) noted that men were significantly more likely to experience negative feelings (e.g., boredom, annoyance) during the bystander training workshop than women.

#### 3.4.2 Organisational outcomes

Six studies reported positive organisational outcomes (Jacobs et al., 2000; Durey, 2011; Stuart et al., 2017; Crisp and Taket, 2018, 2023; Kuppuswami and Ferreira, 2022) while one study reported only negative outcomes (Sarpy et al., 2022). These negative outcomes, resulting from implementation, will be addressed in the section on obstructing factors. Six studies (Bingham and Scherer, 2001; Potter and Moynihan, 2011; Foubert and Masin, 2012; Fischer et al., 2021; Martini and De Piccoli, 2021; Cronin et al., 2024) did not report any organisational outcomes.

##### 3.4.2.1 Cultural shift towards gender equality

Five studies indicated a shift in company culture towards enhanced gender equality and raised awareness of preventing GBVH (Jacobs et al., 2000; Durey, 2011; Stuart et al., 2017; Crisp and Taket, 2018; Kuppuswami and Ferreira, 2022). Crisp and Taket (2018) did not measure organisational outcomes, but anecdotal evidence suggests increased gender equity in the two participating organisations. Both successfully developed and started implementing tailored action plans, though no follow-up was done to determine whether these had any impact. Durey’s (2011) study showed a cultural shift with the company magazine running articles on gender-based violence and employees noting a change in co-worker interactions. Jacobs et al. (2000) reported a significant increase in ‘positive climate’ and cohesion among employees. Kuppuswami and Ferreira (2022, p. 414) found that 50 of 74 participants experienced “an immediate impact at their workplace,” with 16 reporting specific changes like increased gender equality work, promotion of gender-sensitive language, and involving men in creating gender equity. Crisp and Taket (2023) noted that informal feedback suggested the intervention increased leaders’ capacity to model bystander behaviours and respond to violence and abuse.

Stuart et al. (2017) included bystander training as part of a larger gender equality project, making it unclear which outcomes could be directly related to the training. They reported substantial positive changes, such as the creation of gender equity policies, parental leave, and sexual harassment policies, increased promotion of women to leadership, improved relations between men and women, and higher retention of female staff. Two other studies (Durey, 2011; Kuppuswami and Ferreira, 2022) also reported the creation or improvement of gender harassment policies following intervention.

##### 3.4.2.2 Changes in sexual harassment prevalence

Only three studies (Jacobs et al., 2000; Stuart et al., 2017; Fischer et al., 2021) measured the prevalence of sexual harassment, however, Fischer et al. (2021) lacked a baseline for comparison. Jacobs et al. (2000) noted a significant decrease in perceived sexual harassment, gender insensitivity, and gender discrimination from baseline to one-year follow-up. Using retrospective questionnaires and interviews, Stuart et al. (2017) found a decrease in sexual and gender harassment after the intervention.

#### 3.4.3 Retention of effect

While 12 studies indicated some impact from their interventions, only six (Jacobs et al., 2000; Durey, 2011; Potter and Moynihan, 2011; Stuart et al., 2017; Crisp and Taket, 2023; Cronin et al., 2024) employed follow-up measurements. Interestingly, all studies using follow-up metrics were in the ‘successful intervention’ category. Potter and Moynihan (2011) assessed the impact at 1 week and 4.5 months post-implementation but did not share one-week data or compare the two points, leaving retention unclear. The only study (Crisp and Taket, 2023) showing complete retention had questionnaires distributed just 4–6 weeks post-intervention. Therefore, it is uncertain whether sufficient time elapsed to genuinely assess retention. Four studies demonstrated partial retention (Jacobs et al., 2000; Durey, 2011; Stuart et al., 2017; Cronin et al., 2024). Cronin et al. (2024) found that while knowledge and self-efficacy remained significantly higher than baseline, the increase in behavioural intention was sustained only for reporting intentions, while prevention, intervention, and encouragement intention all returned to levels not significantly different from baseline. Durey (2011, p. 53) conducted focus group interviews 6 months post-intervention, reporting that participants had benefited from the training, could recall the “tools to stand up” and found them useful. Jacobs et al. (2000) found that the intervention had reduced perceptions of sexual harassment and decreased the observed instances of such behaviour at a 12-month follow-up. Stuart et al. (2017) reported that some positive changes remained 15 years post-programme, though the impact diminished over the years, partially due to employee turnover. The most lasting impact was personal: “For some, this [GQAL-intervention] was life-changing,” shaping their work, family, and friendships (Stuart et al., 2017, p. 76). This intervention also helped to shift employees’ views on women’s role in society in general. The remaining six studies with positive outcomes did not include follow-up measurements.

### 3.5 Facilitating and obstructing factors for implementation

In the following facilitating and obstructing factors for implementing the interventions across the studies are reviewed.

#### 3.5.1 Facilitating factors

Nine studies reported evaluations of facilitating factors for the implementation of the interventions. In four studies (Stuart et al., 2017; Kuppuswami and Ferreira, 2022; Crisp and Taket, 2023; Cronin et al., 2024) using adaptable interventions tailored to the organisation facilitated successful implementation. Upper management’s commitment and support were reported as crucial for successful GBVH prevention in four studies (Bingham and Scherer, 2001; Stuart et al., 2017; Fischer et al., 2021; Cronin et al., 2024). In three studies (Durey, 2011; Foubert and Masin, 2012; Stuart et al., 2017), framing men as allies facilitated uptake of the intervention message (e.g., being a prosocial bystander) and reduced potential resistance. Finally, an inclusive and participative approach contributed to employment engagement in three studies (Stuart et al., 2017; Crisp and Taket, 2018; Cronin et al., 2024).

#### 3.5.2 Obstructing factors

Eight studies reported evaluation of obstructing factors for implementation of the interventions. Four studies (Durey, 2011; Stuart et al., 2017; Fischer et al., 2021; Kuppuswami and Ferreira, 2022) highlighted issues with intervention delivery or facilitation. A lack of experienced and well-trained facilitators were reported as obstructing factors in two studies (Bingham and Scherer, 2001; Stuart et al., 2017), with Bingham and Scherer’s (Bingham and Scherer, 2001) intervention being designed by employees with no prior experience in sexual harassment prevention which impacted content quality negatively. Other issues included practical considerations like internet access issues (Kuppuswami and Ferreira, 2022), insufficient training capacity (Durey, 2011), and structural complications such as non-mandatory training (Durey, 2011; Fischer et al., 2021) or lack of follow-up when responsible managers were relocated or left the organisation (Stuart et al., 2017). Indeed, lack of managerial support and paucity of resources (e.g., allocated time, personnel) hindered implementation in three studies (Bingham and Scherer, 2001; Naved et al., 2021; Sarpy et al., 2022). This was particularly evident in Sarpy et al. (2022), where these issues and the lack of organisational anchorage led to low employee commitment. Cronin et al. (2024) also reported issues with organisational mistrust, especially among women and underrepresented minority employees. Furthermore, Durey (2011) noted how the organisation failed to understand that cultural changes required a considerable time commitment.

Two studies (Stuart et al., 2017; Naved et al., 2021) showed cultural and political factors hindering implementation. Both studies faced resistance from middle managers, ranging from passive non-corroboration to active sabotage, such as cutting intervention workshops short by several hours (Stuart et al., 2017; Naved et al., 2021) and using threats of violence to make employees falsify survey responses (Naved et al., 2021). Moreover, Stuart et al. (2017) had to halt the implementation due to general strikes and political unrest, while Naved et al. (2021) found that most factories unwillingly participated due to outside pressure from buyers.

## 4 Discussion

This scoping review explored and compared primary-level bystander interventions to prevent gender-based violence and harassment (GBVH), examining their underlying theories, impact, and factors influencing their implementation. A systematic search of five databases yielded 14 eligible studies. Results showed that bystander interventions with training workshops and practice exercises showed greater potential for increasing prosocial bystander behaviour than those relying on passive learning techniques. The most common organisational outcomes were positive changes in culture and climate. Organisational culture represents the enduring values, beliefs, and norms that guide long-term behaviour, while organisational climate captures the current, day-to-day perceptions of the work environment. The most common individual outcomes were increased knowledge about GBVH and improved attitudes towards gender equity and GBVH prevention. Additionally, the results indicated that primary-level bystander interventions should be tailored to the specific workplace context, considering organisational change theories in their development and implementation.

The review showed that SI studies had a broader theoretical framework than PSI studies, supporting the inclusion of theories on bystander behaviour, adult learning theories, intervention theory, and organisational theories in primary-level bystander interventions. This might help explain their higher success rates, yet more dedicated research is needed to establish causality. On the other hand, while interventions in nine of the 14 studies (Jacobs et al., 2000; Durey, 2011; Potter and Moynihan, 2011; Stuart et al., 2017; Crisp and Taket, 2018, 2023; Naved et al., 2021; Kuppuswami and Ferreira, 2022; Sarpy et al., 2022) aimed for organisational change, only three (Durey, 2011; Stuart et al., 2017; Fischer et al., 2021) explicitly included organisational theories in their theoretical foundation.

While the theoretical foundations of interventions are important, their success ultimately depends on how effectively they translate into measurable behavioural change. Only six of the 13 studies aimed at increasing proactive bystander behaviour explicitly measured bystander-related constructs such as behaviours, intention to intervene, or perceived self-efficacy (Potter and Moynihan, 2011; Foubert and Masin, 2012; Fischer et al., 2021; Martini and De Piccoli, 2021; Crisp and Taket, 2023; Cronin et al., 2024). Without these measures, the effectiveness of interventions cannot be fully gauged. Measurements of attitudes towards or knowledge of GBVH can act as proxies but do not reflect behavioural changes.

Most of the included studies did not systematically address the contextual and mediating factors affecting implementation, yet previous research has shown how organisational barriers can diminish the impact of bystander training for both sexual harassment and bullying (Timmerman and Bajema, 2000; Kuntz and Searle, 2023). Indeed, despite sound programme theories, both NSI studies (Naved et al., 2021; Sarpy et al., 2022) experienced issues due to improper consideration of context factors. Thus, when studies of bystander interventions aim for organisational or cultural change, such theories should be included in programme development. Context factors may also affect the long-term efficacy of bystander interventions (Medeiros and Griffith, 2019), underscoring why intervention studies should incorporate these elements. Without a suitable program theory, bystander interventions risk being ineffective or may even increase negative behaviours (as seen in Bingham and Scherer, 2001). Furthermore, failure to report theoretical foundations prevents assessment of whether failures are due to programme or theory failure (Kristensen, 2005). At the same time, recent studies (Coker et al., 2022; Kuntz and Searle, 2023) have also shown that interventions may be successful in the short term but lose impact over time (an issue present in Cronin et al. (2024)), highlighting the need for longitudinal designs. As over half of the studies (8/14) lacked follow-up, our knowledge of the long-term effects of bystander interventions is limited. These issues also apply to organisational outcomes. Ten studies targeted organisational or cultural change, yet only five (Jacobs et al., 2000; Durey, 2011; Stuart et al., 2017; Crisp and Taket, 2018; Kuppuswami and Ferreira, 2022) assessed these factors. Without proper evaluation, it remains unknown if efforts to change organisational culture were successful, risking misdirected efforts and wasted resources.

Another issue is the use of unique scales and constructs instead of validated instruments, complicating comparisons between studies. The challenge of measuring bystander intervention effectiveness is evident when comparing Foubert and Masin’s (2012) study, which separates bystander intentions and efficacy using two different scales, with Martini and De Piccoli’s study, which used the ‘Bystander intention to intervene scale’ but reported results as “Bystander efficacy” (Martini and De Piccoli, 2021, p. 551). This introduces ambiguity about what was measured and complicates comparisons. Furthermore, only three studies (Jacobs et al., 2000; Crisp and Taket, 2018, 2023) measured GBVH prevalence before and after intervention, Cronin et al. (2024) measured number of incident reports, while most studies only measured knowledge or attitudes. Thus, most studies lack direct measures to show whether the interventions had a preventive effect.

A common methodological issue in bystander studies, including those in this review, is the failure to describe whether participants had the chance to employ bystander behaviours (Kistler et al., 2022). Without controlling for this factor, it is difficult to determine if interventions fail or if participants lack opportunities to use new skills. This highlights a long-standing debate on measuring bystander behaviours and assessing intervention effectiveness (for further discussion see Hoxmeier et al. (2023)). Our findings show how these methodological issues complicate primary bystander intervention research, evaluation and program development.

### 4.1 Relevance and recommendations for practise

Our review highlights diverse methods for constructing and implementing successful GBVH bystander training. Findings pointed to active learning, especially practice exercises, proving superior to passive learning (e.g., lectures). Merely educating people about GBVH may thus be insufficient. Instead, interventions should include opportunities to practise bystander behaviours, such as roleplay. Moreover, even though both single-session, multi-session, and complex multicomponent interventions showed some success, we recommend the latter. GBVH has complex and multifaceted antecedents, therefore prevention efforts should be a holistic, whole-of-company project (Willness et al., 2007). This aligns research on bystander interventions for other types of workplace mistreatment such as bullying and incivility (Hershcovis et al., 2020; Pouwelse et al., 2021), sexual assault in higher education (Kettrey and Marx, 2021; Coker et al., 2022), and with Bell et al.'s (2002) ‘best practise’ guidelines for GBVH intervention, which also stresses how organisational change requires consistent efforts. They propose a multicomponent intervention with four key elements: (1) developing a zero-tolerance policy, (2) displaying the policy in multiple places in the organisation, (3) regular and directed training (e.g., bystander training), and (4) securing commitment from top management. Recent studies (Medeiros and Griffith, 2019; Lassiter et al., 2021) support these recommendations. Indeed, three of the four components recommended by Bell et al. (2002), regular bystander training, zero-tolerance policies, and leadership support, were found, in part, to be common factors between SI and PSI, although none of the included studies incorporated all four.

Building on our findings, we propose that organisations and practitioners prioritise designing or using interventions grounded in sound and comprehensive programme theory. This should include known antecedents of GBVH (e.g., unequal power relations (O’Connor et al., 2021)), theories on adult learning, bystander behaviour theory, and organisational change theories including known contextual and cultural factors. A comprehensive organisation-specific needs assessment should also be part of the development phase (Medeiros and Griffith, 2019).

### 4.2 Future research

We have identified several promising avenues for future research on primary bystander interventions. First, using longitudinal research designs to investigate long-term effects is crucial. Research with clear, consistent outcome measures and both baseline and follow-up measures of targeted behaviour prevalence would facilitate easier comparisons across studies. These designs should use multiple measurements at fixed time points, with and without additional interventions, to determine if different recurring training types better retain positive outcomes (cf. Coker et al., 2022). This could help organisations decide on training type and frequency. Second, better methods are needed to distinguish between different bystander constructs (e.g., intent and efficacy) and to separate a lack of displayed bystander behaviours from a lack of opportunities to do so. Mixed method research designs could achieve this by using surveys on bystander intentions and actions, along with in-depth interviews uncovering participants’ experiences [for an example see Nielsen et al., 2025]. Third, using mixed methods future studies should examine the role of contextual factors when implementing primary bystander interventions (see also Nielsen and Abildgaard, 2013; Nielsen et al., 2025). Related to this, organisational and supra-organisational factors such as national cultures significantly influence the interpretation of social situations (Brislin, 1993), leading to variations in personal boundaries and definitions of inappropriate workplace behaviour across countries (Yee et al., 2015) and organisations (Benavides Espinoza and Cunningham, 2010). Though the included studies were conducted across different sectors, we did not assess whether industry characteristics affected intervention outcomes. This would be an interesting avenue to pursue further in future research. Despite the diverse range of workplaces, the studies were mostly from Western cultures, and none of them addressed intersectionality, i.e., how multiple stigmatised social identities (e.g., gender, sexuality, race) interact to increase the risk of GBVH (Collins, 2015). This limitation should be addressed in future research (McDonald, 2012; UN Women and International Labour Organization, 2019).

### 4.3 Limitations

The present review is not without limitations. Capturing the diversity in nomenclature for bystander interventions aimed at preventing GBVH is challenging. We attempted to address this with a nuanced and wide-ranging search strategy. Another limitation is the vast differences in intervention designs and outcome measures, making it difficult to compare interventions directly, leading to an analysis on a more general level.

Furthermore, we only found 14 bystander intervention studies with varied research quality. Using Murphy’s (1996) five-point rating system for research designs only Foubert and Masin (2012) qualified for a five-star rating (properly conducted studies with randomised control groups, while Cronin et al. (2024) were in the four-star category (properly conducted studies with control groups but without randomisation). Most studies used a non-randomised experimental design without control groups or randomisation (3-star category), which is seen as the minimum acceptable standard (Kompier et al., 1998). Three studies reporting organisational outcomes relied on anecdotal or descriptive evidence (Durey, 2011; Crisp and Taket, 2018; Kuppuswami and Ferreira, 2022), which is the lowest quality of evidence. As such, caution must be advised when interpreting the findings.

## 5 Conclusion

The present study is the first scoping review seeking to provide valuable insights into primary-level bystander interventions addressing gender-based violence and harassment (GBVH) in the workplace. The mapping of the 14 identified studies revealed a wide spectrum of interventions with the most common being complex multicomponent interventions. Bystander interventions with training workshops and practice exercises showed greater potential for increasing prosocial bystander behaviour than interventions relying on passive learning techniques. Complex interventions with multiple components and diverse content delivery strategies generally outperformed single-session interventions. The study pointed to variations in the studies’ theoretical fundament and that the employment of comprehensive frameworks incorporating bystander behaviour, violence prevention, and adult learning theories appeared more effective. Corroborating results from organisational intervention research, implementation success also appeared to rely on whether contextual and cultural factors were addressed. Despite a general lack of systematic assessments of mediating contextual factors in the studies, leadership commitment and support emerged as crucial facilitating factors. Likewise, lack of leadership support and commitment and practical difficulties in delivering and implementing interventions acted as obstructing factors. In conclusion, our findings support the use of primary-level bystander interventions against GBVH in the workplace, yet more high-quality research is needed to determine which intervention designs and delivery methods work best.

## Data Availability

The original contributions presented in the study are included in the article/Supplementary material, further inquiries can be directed to the corresponding author/s.
