# Supplementary material for: Bystander interventions against gender-based violence and harassment in the workplace: a scoping review
Source: Front Psychol. 2025 May 29;16:1570812. doi: 10.3389/fpsyg.2025.1570812 (PMC12161231; doi:10.3389/fpsyg.2025.1570812)
Supplement: Supplementary file 1 [file Data_Sheet_1.DOCX]

Contents

[Search strings 2](#_Toc176954854)

[Data extraction form 3](#_Toc176954855)

# Search strings

**Search string used in OVID databases (PsycINFO, Embase, MEDLINE):**

((sex* adj7 harass*) or (emotion* adj7 abuse) or (gender* based adj7 harass*) or (gender* adj7 harass*) or unwant* sex* attention* or sex* coercion* or (gender* based adj7 violence*) or gender* violence* or (gender* based adj7 aggressi*) or gender* equ* or sexi* or gender* discrimi* or (sex* adj7 bully*) or (sex* adj7 descrimin*) or gender* related discrimi*) AND (risk reduc* OR prevent* OR reduc* OR counteract* OR resist* OR hinder* OR action*) AND (organi#ation* OR workplace* OR work* place* OR jobsite OR job site* OR work environment OR work* condition* OR job OR employme* OR universit* OR Colleg*) NOT (child* OR kid*).ti

**Search string used in SCOPUS:**

TITLE-ABS-KEY(((sex* harass*) OR (emotion* abuse*) OR (gender* based* harass*) OR (gender* harass*) OR (unwant* sex* attention*) OR (sex* w/4 coercion*) OR (gender* based* violenc*) OR (gender* violence) OR (gender* based* aggressi*) OR (gender* equ*) OR sexis* OR (gender* discrimi*) OR (sex* bully*) OR (sex* discrimi*) OR (gender* relat* discrimi*)) AND ((risk reduc*) OR prevent* OR reduc* OR counteract* OR resist* OR hinder* OR action*) AND (organi?ation* OR workplace* OR (work* place*) OR jobsite OR (job site*) OR (work environment) OR (work* condition*) OR job OR employme* OR universit* OR Colleg*) AND (interven* OR initiativ* OR implemen* OR (employe* program*))) AND NOT TITLE(kid* OR Child*)

**Search string used in Web of Science:**

TS=(((sex* harass*) OR (emotion* abuse) OR (gender* based harass*) OR (gender* harass*) or (unwant* sex* attention*) OR (sex* coercion*) OR (gender* based violence*) OR (gender* violence*) OR (gender* based aggressi*) OR (gender* equ*) OR sexi* OR (gender* discrimi*) OR (sex* bully*) OR (sex* descrimin*) OR (gender* related discrimi*)) AND ((risk reduc*) OR prevent* OR reduc* OR counteract* OR resist* OR hinder* OR action*) AND (organi?ation* OR workplace* OR (work* place*) OR jobsite OR (job site*) OR (work environment) OR (work* condition*) OR job OR employme* OR universit* OR Colleg*) AND (interven* OR initiativ* OR implemen* OR (employe* program*))) NOT TI=((child* OR kid*))

# Data extraction form

**General information**

- Citation
- Title of the article
- In which country was the study conducted?

**Characteristics of the study**

- Methods:
  - Aim of the study
  - Expected outcomes or hypotheses
  - Study design
    - Randomised controlled trial
    - Non-randomised experimental study
    - Cohort study
    - Cross sectional study
    - Case control study
    - Qualitative research
    - Other
  - Type of comparator
    - No comparator
    - No intervention (standard practice)
    - Active comparator
    - Other
  - Measurement instrument
- Intervention characteristics
  - Brief description of intervention
  - Theoretical foundation
  - Intervention aim
  - Number and type of interventions (including details like session length etc.)
  - Method of delivery (e.g., in person or online)
  - Presenter/educator (peer-presenter, trained staff, the researcher etc.)
  - Target audience
- Participants
  - Participant sex
    - Male-only
    - Female-only
    - Mixed group
  - Total number of participants
- Outcome measures and results
  - Individual outcome measures reported
  - Organisational outcome measures reported
  - Retention of effect (follow-up study)
  - Factors facilitating implementation of the intervention
  - Factors obstructing implementation of the intervention
